# Characterization of Algae Dietary Supplements Using Antioxidative Potential, Elemental Composition, and Stable Isotopes Approach

**DOI:** 10.3389/fnut.2020.618503

**Published:** 2021-02-05

**Authors:** Jan Kejžar, Marta Jagodic Hudobivnik, Marijan Nečemer, Nives Ogrinc, Jasmina Masten Rutar, Nataša Poklar Ulrih

**Affiliations:** ^1^Department of Food Science and Technology, Biotechnical Faculty, University of Ljubljana, Ljubljana, Slovenia; ^2^Department of Environmental Sciences, Jožef Stefan Institute, Ljubljana, Slovenia; ^3^Department of Low and Medium Energy Physics, Jožef Stefan Institute, Ljubljana, Slovenia

**Keywords:** algae, *Spirulina*, *Chlorella*, *Aphanizomenon flos-aquae*, antioxidative potential, stable isotopes, elemental composition, toxic elements

## Abstract

Dietary supplements based on algae, known for their nutritional value and bioactive properties, are popular products among consumers today. While commercial algal products are regarded safe by numerous studies, information about the production and origin of such products is scarce. In addition, dietary supplements are not as strictly regulated as food and medicinal drugs. We characterized different algal products (kelps: Laminariales, *Spirulina* spp., *Chlorella* spp., and *Aphanizomenon flos-aquae*), obtained on Slovenian market, based on their elemental composition (X-ray fluorescence, inductively coupled plasma–mass spectrometry), antioxidative potential [DPPH (2,2-diphenyl-1-picrylhydrazyl) assay, total phenolic content], and stable isotope values [carbon (C), nitrogen (N), and sulfur (S); elemental analyzer isotope ratio mass spectrometry (EA-IRMS) method]. Antioxidative potential is consistent among products of the same type, with *A. flos-aquae* samples having 4.4 times higher antioxidative potential compared to *Chlorella* spp. and 2.7 times higher compared to *Spirulina* spp. Levels of toxic trace elements (arsenic, cadmium, mercury, and lead) are below the maximum allowed values and as such do not pose risk to consumers' health. Samples of *Spirulina* spp. have relatively high δ^15^N (7.4 ‰ ± 4.4‰) values, which indicate use of organic nitrogen sources in certain samples. Likewise, different elemental composition and isotopic ratios of stable elements (C, N, and S) for the samples with *Spirulina* spp. or *Chlorella* spp. are the consequence of using different nutrient sources and algae-growing techniques. Statistical analysis (principal component analysis) has confirmed that all tested *A. flos-aquae* samples originate from the same source, supposedly Klamath Lake (Oregon, USA). Hawaiian *Spirulina pacifica* can also be differentiated from all the other samples because of its characteristically high metal content (iron, manganese, zinc, cobalt, nickel, vanadium). *Chlorella* spp. and *Spirulina* spp. require further analyses with larger number of samples, as differentiation is not possible based on results of this study.

## Introduction

There are numerous algae-based dietary supplements available on the market, which indicates their widespread use among consumers. Dietary supplements are not subject to strict regulations like drugs and imported food. Therefore, continuous evaluation of efficacy, safety, and origin is required to guarantee quality of dietary supplements. Comparison between different products is also complicated because of addition of unknown compounds, which is a common practice among manufacturers. Microalgae (unicellular eukaryotes and cyanobacteria) are interesting organisms to cultivate because of their ability to synthesize bioactive compounds and accumulate minerals and high nutritional value. They are able to grow in modified mediums, including wastewater, which additionally improves economic viability of cultivating microalgae (1). Currently, most producers of microalgae-based commercial products are located in Asia or Australia and show an impressive growth. Production share of food/feed microalgae products owned by European companies is estimated to be approximately 5% of the global market (2).

Microalgae-based products (*Spirulina* spp.—*Arthrospira* spp., *Chlorella* spp., and *Aphanizomenon flos-aquae* or AFA) have the highest market share among “algal” dietary supplements. *Spirulina* spp. products, in tablet or powder form, are mostly consumed because of their nutritional profile: protein (60–70%), carbohydrates (14–19%), fat (8%), dietary fibers (3%), vitamins (<1%), and phytochemicals (3, 4). Algae products are also regarded as a significant source of major elements, such as iron (Fe), calcium (Ca), phosphorus (P), potassium (K), sodium (Na), and magnesium (Mg), and trace elements, including manganese (Mn), zinc (Zn), copper (Cu), selenium (Se), and chromium (Cr). Recommended daily amount of aforementioned algae can provide substantial amount of these minerals and even fulfill recommended dietary allowance for iron intake (4, 5). Studies on content of toxic elements and cyanotoxins are scarce (6). Studies (7–10) done on safety of microalgae do not necessarily reflect safety of algal products, as commercial cultivation practice is unknown and not subject to strict regulations. Results acquired from laboratory grown algae are potentially misleading as different growing conditions significantly impact the content of certain elements and synthesized metabolites in algae (1). Determining efficacy and safety of algal products therefore requires analysis and comparison of individual samples from different manufacturers.

Manufacturers provide little to no information regarding the origin and manufacturing practices of their algae-based dietary supplements. Nutrient composition and toxic compounds differ, depending on the location of sample production. Reasons are various environmental conditions and agrotechnical measures. Thus, to ensure quality and safety of the products used in daily nutrition, determination of product's origin is of great importance (11). Variable environmental conditions that influence microalgal growth can consequently affect the stable isotopic composition of carbon, nitrogen, and sulfur (C, N, and S). These parameters along with elemental composition could be used to verify the quality and origin of microalgal products.

The aim of our study is to differentiate commercially available algal products on Slovenian market by characterizing them based on antioxidative potential, elemental composition and stable isotope composition of C, N, and S, as they can reflect different growing conditions, sources of nutrients and origin. To our knowledge, such an approach has not yet been used in any previous study of algae-based supplementary products.

## Materials and Methods

### Sample Collection

In the present study, 18 samples were obtained from several physical stores and web stores in Slovenia (2018). Dietary supplements were selected based on several types of algae [kelps: Laminariales (*n* = 2), *Spirulina* spp. (*n* = 7), *Chlorella* spp. (*n* = 5), and AFA (*n* = 4)] with different types of production—conventional and organic. The samples were intended for sale on Slovenian market and have declaration in Slovene language (Table 1).

**Table 1:** List of algae-based dietary supplement samples obtained on Slovenian market with the information on purity, origin, and suggested daily use.

**Algae**	**Sample**	**Purity**	**Origin**	**Declared growing practice**
*Laminaria digitata* and *Ascophyllum nodosum*	S1	Additives	Not specified	Conventional
*Macrocystis pyrifera*	S5	Additives	Not specified	Conventional
*Aphanizomenon flos-aquae*	S2	Additives	Klamath Lake	Conventional
*A. flos-aquae*	S10	Pure	Klamath Lake	Organic
*A. flos-aquae*	S11	Pure	Klamath Lake	Conventional
*A. flos-aquae*	S12	Additives	Klamath Lake	Conventional
*Chlorella pyrenoidosa*	S3	Pure	Not specified	Conventional
*Chlorella* sp.	S4	Pure	China	Conventional
*Chlorella* sp.	S7	Additives	Not specified	Conventional
*Chlorella vulgaris*	S8	Pure	Outside of EU	Organic
*Chlorella* sp.	S9	Pure	Outside of EU	Organic
*Spirulina platensis*	S6	Pure	Not specified	Conventional
*Spirulina pacifica*	S13	Additives	Hawaii	Conventional
*Spirulina* sp.	S14	Pure	Outside of EU	Organic
*S. platensis*	S15	Pure	Outside of EU	Organic
*S. platensis*	S16	Pure	Taiwan	Organic
*S. pacifica*	S17	Additives	Hawaii	Conventional
*Spirulina maxima*	S18	Additives	Italy	Conventional

### Sample Preparation

Samples in tablet form were ground to fine powder, and samples in capsules were opened. All samples were subsequently stored in powdered form in plastic containers with screw caps. During analysis, all samples were stored at room temperature and kept away from direct sunlight, following the manufacturers' storage guidelines. Sample preparation step was repeated for each individual analysis.

### Sample Extract Preparation for Determination of Total Phenolic Compounds and Antioxidative Potential

Five hundred milligrams of fine powder sample was added to 10 mL 80% methanol solution in a centrifuge tube. After vortex mixing it for 5 min, it was incubated in ultrasound bath for 30 min at 40°C. Following incubation, samples were centrifuged for 20 min at 9,400 rcf (at 20°C) and filtrated through filters with 0.32-μm pore width into centrifuge tubes. Ten milliliters of 80% methanol solution was added to resulting sample sediment, and the whole procedure was repeated for each sample. We added 80% methanol solution until 20-mL volume was reached. Sample extracts were prepared in duplicate. Extract of each duplicate sample was stored in four vials, containing 5 mL extract each (total 20 mL per sample duplicate) at −20°C.

### Total Phenolic Content

The total phenolic content (TPC) of algal methanolic extracts was determined by Folin–Ciocalteu method (12). Twenty milliliters of Folin–Ciocalteu reagent solution was prepared by mixing MilliQ water (resistivity of 18.2 MΩ^*^cm (at 25°C) and total organic C value <5 ppb) in ratio 1:10. Mixture in screw cap tube was prepared by adding 0.2 mL Folin–Ciocalteu solution, 0.2 mL sufficiently diluted methanolic sample extract, 1 mL Na carbonate solution (mass concentration of 75 g/L), and 2 mL MilliQ water. After thorough vortex mixing, the mixture was incubated for 2 h in the dark at room temperature, followed by 5-min centrifugation at 2,000 RPM.

Spectrophotometer was calibrated using blind sample. The sample was prepared in the same way as other samples, except that 0.2 mL of 80% methanol was added instead of 0.2-mL sample extract. All measurements were performed at wavelength of 750 nm. Sample dilution ratio was determined for each individual sample by test runs using the same procedure. Kelp samples' total phenolic compounds content was too low to be detected by our method (even after the manipulation of sample dilution ratio in final mixture). Calibration curve for TPC analysis was prepared with gallic acid in triplicate with concentrations 5, 10, 15, and 20 mg/mL. Gallic acid solutions were prepared according to the same protocol as samples, in 80% methanol solution. Results were expressed as mg gallic acid equivalent (GAE)/g solid sample mass.

### DPPH Assay

The free radical–scavenging activity of algal extracts were measured by the decrease of absorbance of methanolic solution of 2,2-diphenyl-1-picrylhydrazyl (DPPH) (13). A stock methanolic solution of DPPH (0.0837 μM) was prepared by mixing 3.3 mg DPPH in 100 mL of pure methanol. Absorbance of stock DPPH solution was ~1.1 at 517 nm. Final mixture was prepared by mixing 0.5 mL of sufficiently diluted sample extract and 2.5 mL of methanolic DPPH solution. Blank samples were prepared with 0.5 mL diluted sample extract and 2.5 mL pure methanol. Control sample was prepared with 0.5 mL pure methanol and 2.5 mL methanolic DPPH solution. Absorbance was measured after 30-min incubation period at room temperature in the dark.

Sample dilution ratio was determined for each individual sample by test runs using the same procedure. Antioxidative potential of kelp samples was below detection of our method and could not be determined. On the contrary, AFA samples required dilution in ratio 1:5. Calibration curve for DPPH analysis was prepared with gallic acid in triplicate with concentrations 6, 4.5, 3, 1.5 and 0.75 μg/mL. Like samples, gallic acid was prepared in 80% methanol solution.

### Elemental Composition Determination Using X-Ray Fluorescence

X-ray fluorescence (XRF) analysis was performed at Jožef Stefan Institute, Ljubljana. Powdery samples were pressed into tablets. Quality assurance for element analysis was performed using standard reference materials 1573A National Institute of Standards and Technology (NIST) tomato leaves and 1547 NIST peach leaves, acquired from the NIST (Gaithersburg, MD, USA). XRF was used to determine the following elements: bromine (Br), calcium (Ca), chlorine (Cl), iron (Fe), iodine (I), potassium (K), manganese (Mn), phosphorus (P), rubidium (Rb), sulfur (S), silicon (Si), strontium (Sr), titanium (Ti), and zinc (Zn).

### Sample Preparation for Inductively Coupled Plasma–Mass Spectrometry

Samples were digested using an UltraWAVE digestion system (Millestone, Italy); 0.05–0.1 g of sample was weighted directly into Teflon vial, followed by addition of 2 mL of 65% HNO_3_ (Suprapur, Merck, Germany). Digestion temperature program was as followed: from room temperature to 240°C in 20 min, held on 240°C for 15 min, and then cooled to 40°C (approximately 1 h). Maximum pressure was set at 100 bar. Loading gas (N_2_) was set at 25 bar and room temperature.

Digested samples were transferred into plastic vials and filled to 10-mL mark with MilliQ water. Samples were additionally diluted with 5% HNO_3_ in 1:5 ratio. Because of visible residuals, the samples were filtered through 0.45-μm hydrophilic syringe filters (Millipor Millex-HV, Merck, Germany). Quality assurance for element analysis was performed using standard reference material BCR-414 (trace elements in plankton) with known elemental composition. Reagents' blanks were prepared according to the same protocol as samples.

### Elemental Composition Determination Using Inductively Coupled Plasma–Mass Spectrometry

Inductively coupled plasma–mass spectrometry (ICP-MS) analysis was performed on an Agilent 8800 triple quadrupole instrument (Agilent Technologies, California, USA). Calibration curve for mercury (Hg) content determination was prepared using NIST 3133 Hg standard solution in the following concentrations: 0, 0.1, 0.5, 1, and 5 ng/mL. Elements [V, Mn, Co, Ni, Cu, Zn, As, Se, Sr, Mo, Cd, Pb and Fe] were determined using MULTI XVI (Merck, Germany) multielement standard solution for ICP-MS. Calibration curve was prepared in 5% HNO_3_ by using the following concentrations of MULTI XVI: 0, 0.1, 0.5, 1, 5, 10, 50, 100, and 250 ng/mL.

Each sample was analyzed in dilutions (with 5% HNO_3_) to 10 and 100 mL. Samples S1, S4, S7, and S10 were prepared in duplicate (two parallels for 10-mL dilution, two parallels for 100-mL dilution). ICP-MS was used for determination of following trace elements: As, Cd, Co, Cu, Hg, Mn, Mo, Ni, Pb, Se, Sr, V, and Zn.

### Stable Isotope Ratio Analysis of Light Elements Using EA-IRMS

Stable isotope ratios of ^13^C/^12^C, ^15^N/^14^N, and ^34^S/^32^S were expressed as δ values in ‰ according to the following equation (14):

(1) δijE=Rijsample−RijrefRijref

where E represents element (C, N, S), R is isotope ratio between heavier “*i*” and lighter “*j*” isotopes (^13^C/^12^C, ^15^N/^14^N, ^34^S/^32^S) in the “sample” and reference material (“ref”). Values for C were expressed relative to V-PDB (Vienna-Pee Dee Belemnite) standard, N values relative to AIR, and S values relative to V-CDT (Vienna Cañon Diablo Troilite) standard.

Stable isotope ratios of light elements (^13^C/^12^C, ^15^N/^14^N, ^34^S/^32^S) in algae samples were simultaneously determined by isotope ratio mass spectrometry with preparation system for solid samples IsoPrime 100–Vario PYRO Cube (OH/CNS Pyrolyser/Elemental Analyzer, Elementar Analysensysteme GmbH, Germany). Four milligrams of sample and 4 mg of tungsten oxide (WO_3_) were weighted directly into tin capsules, sealed, and placed into the automatic sampler of the elemental analyzer. Each sample was measured in triplicate, and the average value was considered. Quality assurance for stable isotope ratio analysis was performed using the following reference materials: USGS-43: δ^13^C = −21.28 ± 0.10‰, δ^15^*N* = +8.44 ± 0.10‰, δ^34^S = +10.46 ± 0.22‰; B2155 Protein Sercon: δ^13^C = −26.98 ± 0.13‰, δ^15^*N* = +5.94 ± 0.08‰, δ^34^S = +6.32‰ ± 0.8‰; Casein Protein CRP: δ^13^C = −20.34 ± 0.09‰, δ^15^*N* = +5.62 ± 0.19‰, δ^34^S = +4.18‰ ± 0.74‰. Measurement precision value was ±0.2‰ for δ^13^C, and ± 0.3‰ for δ^15^N and δ^34^S.

### Statistical Analysis

Statistical calculations were carried out using the XL-STAT software package (Addinsoft, Long Island, NY, USA, 2019). First, basic statistics were applied to the data. Because most of the data were not normally distributed (Shapiro-Wilk test, *p* < 0.05), the nonparametric Mann–Whitney *U* test was used for comparison of element content between different microalgae products. In all analyses, *p* < 0.05 was considered as statistically significant.

Further, principal component analysis (PCA) was applied. PCA is an unsupervised pattern recognition multivariate statistical tool able to analyze numerical dataset structured in an M observations/N variables table and recognize underlying patterns in the dataset. The results of such analysis are displayed as biplots, which are simultaneous representations of variables and observations in the space of selected two PCA axes (e.g., PC1/PC2). The biplots enable visualization and increase interpretability of relation and trends among observations and variables on a two-dimensional map and identify similarities and differences among observations, association with variables, and also impact and role of a particular variable in discrimination and clustering of observations.

PCA was used to enable identification of characteristic parameters that are able to discriminate samples based on antioxidative potential, stable isotope composition of light elements and elemental composition. Samples of kelps (Laminariales) were excluded from PCA, because of relatively low content of algae in dietary supplement products and significant physiological differences compared to microalgae.

## Results and Discussion

### Total Phenolic Content and Antioxidative Potential

**Figure 1:**
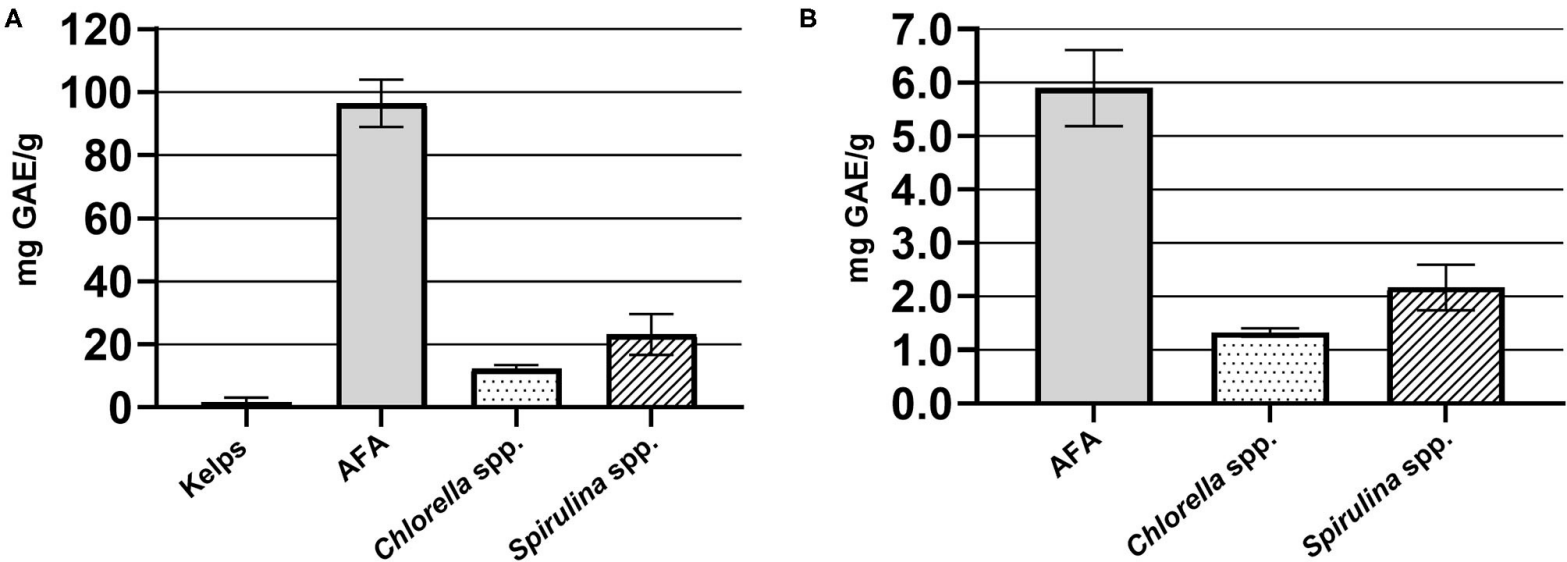
**(A)** Average total phenolic compounds (Folin–Ciocalteu method); **(B)** average antioxidative potential values (DPPH method) in different types of algae-based dietary supplement samples, expressed as mg gallic acid equivalent (GAE)/g solid sample.

Average values of TPC of different dietary supplements based on algae, expressed as mg GAE/g solid sample, were 12.3 ± 1.2 for samples of *Chlorella* spp., 23.2 ± 6.4 for *Spirulina* spp., 96.4 ± 7.5 for AFA, and 1.6 ± 1.6 for kelp (Figure 1). AFA samples contained the highest amounts of TPC, followed by *Spirulina* spp. and *Chlorella* spp. For comparison, Al-Dhabi and Valan Arasu (15) determined TPC values of 2.4–24.4 mg GAE/g sample for *Spirulina* spp. Kelp samples had negligible TPC content, presumably due to the low algae content in the sampled product itself (13–14% according to the declaration). It should be noted that kelp samples were not homogenous (brown algal parts tabletized with filler); therefore, the measurements might be incorrect.

Average antioxidative potential values, determined by the DPPH method, for different types of algae-based dietary supplement samples (expressed as mg GAE/g solid sample) were 1.33 ± 0.08 for *Chlorella* spp., 2.17 ± 0.43 for *Spirulina* spp., and 5.90 ± 0.71 for AFA (Figure 1). Antioxidative potential of kelp was significantly lower in comparison to other algae samples and as such could not be measured by using our method.

AFA samples showed the highest measured antioxidative potential, which was 4.4 times the value of *Chlorella* spp. samples and 2.7 times the value of *Spirulina* spp. samples. There were no significant differences among similar products from different manufacturers regarding antioxidative potential. The latter is evident from the relatively small deviations in measured values among sample groups with the same algae type (Figure 1). Consequently, antioxidative potential allows discrimination of samples between different algae types. It should be noted that presented results of antioxidative potential do not assess the efficacy in relation to health benefits of algal products, as our research goal is mainly characterization of different product types.

### Stable Isotope Composition of Light Elements

δ^13^C value in algae is reflected by their C source (16). Average δ^13^C value of *Spirulina* spp. samples was −23.0‰ ± 4.0‰. Unusually high δ^13^C value of −17.4‰ of sample S18 could be explained by declared addition of corn maltodextrin, which has characteristic δ^13^C value of C4 plants (from −15‰ to −12‰) (17). *Chlorella* spp. had similar δ^13^C values with an average value of −27.5‰ ± 5.7‰, with sample S3 having the lowest value of −37.1‰ (Table 2). The low δ^13^C value determined in *Chlorella* spp. could be related to the growing conditions in a closed system. Closed systems are closed bioreactors with higher control over growing conditions (pH and temperature), higher photosynthetic efficiency, lower water evaporation rate, and lower CO_2_ loss to the atmosphere (18). It was found that the δ^13^C value in a closed system exhibits lower δ^13^C value of CO_2_. Because of relatively small deviation among samples of *Spirulina* spp. and *Chlorella* spp., we assume they utilize similar sources of ${\text{HCO}}_{3}^{-}$ and CO_2_ during cultivation, presumably ones that have shown to be the most efficient from manufacturer's point of view.

**Table 2:** δ^13^C, δ^15^N and δ^34^S values in algal dietary supplement samples.

**Algae**	**Sample**	**δ^**13**^C_**V−PDB**_ (‰)**	**δ^**15**^N_**AIR**_ (‰)**	**δ^**34**^S_**V−CDT**_ (‰)**
Kelp	S1	−25.5	8.8	15.5
	S5	−22.1	10.3	16.8
*Aphanizomenon flos-aquae*	S2	−16.8	1.8	4.9
	S10^a^	−15.4	−0.5	5.1
	S11	−15.8	0.4	5.3
	S12	−14.1	−0.6	5.3
*Chlorella* spp.	S3	−37.1	−0.7	1.7
	S4	−25.9	6.6	−0.9
	S7	−25.7	3.4	−3.0
	S8^a^	−22.0	−3.4	1.3
	S9^a^	−27.0	11.8	−0.9
*Spirulina* spp.	S6	−27.7	1.2	−0.2
	S13	−25.8	10.8	8.8
	S14^a^	−26.1	8.8	11.3
	S15^a^	−22.1	7.6	−0.6
	S16^a^	−21.8	6.2	11.5
	S17	−24.4	13.8	7.8
	S18	−17.4	13.3	11.0

a *Declared as organic*.

*Spirulina* spp. samples had an average δ^15^N value of 7.4‰ ± 4.4‰, indicating organic source of N in samples with high δ^15^N values (>6‰), possibly due to wastewater use (19). This also applies to kelp samples. Higher δ^15^N values also indicate usage of modified mediums for algae cultivation, as the latter can significantly improve economic viability of the project. One sample (S6) of *Spirulina platensis* differed from other samples with δ^15^N value of 1.2‰, indicating inorganic source of N or molecular N (air) fixation. Differences in δ^15^N values between samples can also be explained by other factors, such as (i) different climate, which is hard to control in open bioreactors, and (ii) recycling of growth medium (1). Samples of *Chlorella* spp. generally showed lower δ^15^N values (3.5 ± 6.0‰) compared to *Spirulina* spp. samples. This is probably due to *Chlorella* manufacturers using less optimized and modified growing methods compared to *Spirulina* manufacturing. *Spirulina* is able to grow in saline environments (8), which is exploited to prevent contamination by other microorganisms when growing in “low-quality” media. As original media use mostly inorganic source of N (1), we can assume that most samples were grown using modified media. Lack of information from manufacturers makes interpretation of results rather difficult, as we do not have any insight into geographical factors that may affect fractionation.

AFA samples had similar stable isotope composition of C and N, which indicates that samples originate from the same source (all AFA products are declared to originate from Klamath Lake, OR). Small deviations in stable isotope composition of C and N in sample S2 were probably due to presence of additives in the product. Relatively high values of δ^13^C (−15.5 ± 1.1‰) in AFA samples might indicate photosynthetic fixation of CO_2_ from air as their primary source of C. Values of δ^15^N were around zero (0.3 ± 1.1‰), indicating fixation of molecular N from air.

The δ^34^S values in algae-based dietary supplement samples ranged from −3.0 to 16.8‰, with the lowest values observed in *Chlorella* spp. and the highest in kelps (brown algae). Variability in δ^34^S values among the samples of same algae species (with the exception of AFA) was on average higher for C and N. This variability can be explained by different origins of samples or differences in organic load during growing conditions. However, the information regarding the distribution of the δ^34^S values of aquatic resources and organisms is scarce. There are three potential sources of S in algae, depending on the proximity to the ocean, geology, and redox chemistry. For example, the δ^34^S of marine sulfate and vegetation near the ocean are approximately +20‰ but decrease to +6‰ over 100 km (20, 21). In the Hawaiian Islands, δ^34^S values of sulfates from volcanic ash and basalt-derived soils ranging from 6.3 to 15.4‰ (22) have been reported and are also in agreement with our data.

### Elemental Analysis

The results of the elemental composition in microalgae supplements are presented in Supplementary Table 1. No statistically significant difference between different types of algae supplements was observed for Si, V, Mn, Ti, Co, Ni, Rb, Cu, Se, Mn, Fe, and Hg. A significantly higher content of Ca was observed for *Spirulina* spp. products, whereas *Chlorella* spp. displayed the highest P level. This observation agrees with the study performed by Rzymski et al. (7). AFA products exhibited high Ca and Mo concentrations that differ statistically significantly from other products. The highest concentrations of Sr, Br, and Cl were determined in kelps samples and differ significantly from other products mainly from *Chlorella* spp. Zn levels did not differ between *Spirulina* spp. and *Chlorella* spp., but they are significantly higher than those observed in AFA and kelps.

### Iron Content

**Figure 2:**
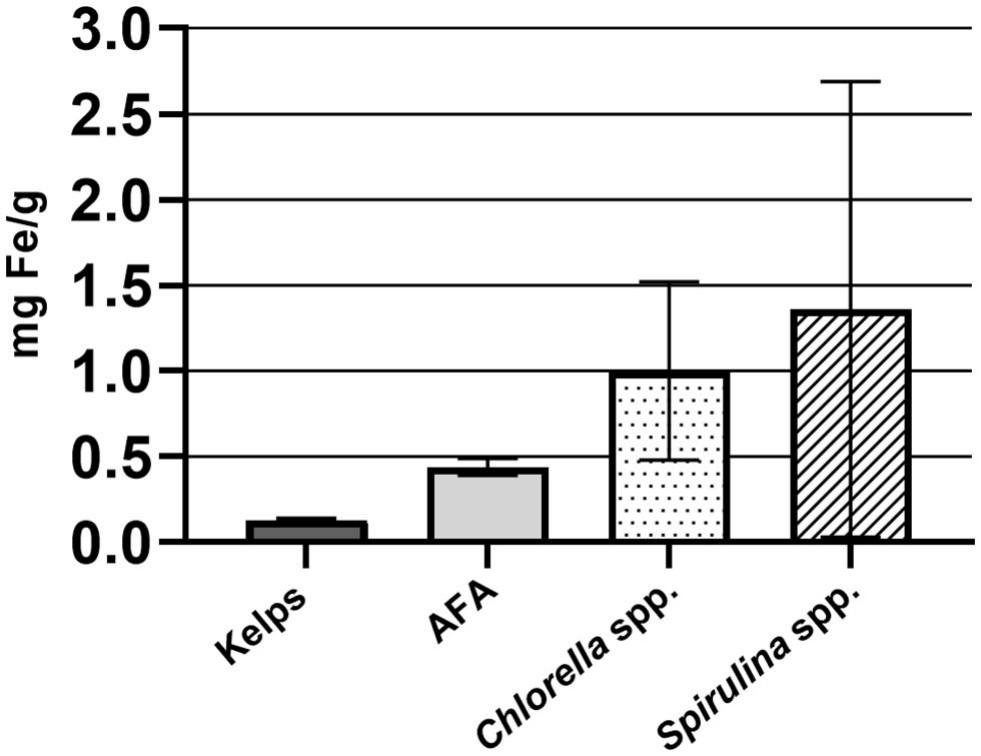
Average iron content in algal dietary supplement samples, expressed as mg iron/g solid sample.

Average Fe content (mg Fe/g solid sample) in samples of *Chlorella* spp. was 1.00 ± 0.52, *Spirulina* spp. 1.36 ± 1.33, AFA 0.44 ± 0.05, and kelps 0.13 ± 0.01 (Figure 2). High deviation among *Spirulina* spp. samples is due to higher Fe content in Hawaiian *Spirulina pacifica* samples (3.29 ± 0.27). Iron content in *Spirulina* reflects the Fe content in the medium used for cultivation (23).

### Iodine Content

Iodine content in kelp samples was 183 mg/kg solid sample for S1 and 221 mg/kg for S5, with 3% and 11% deviation from values declared on the product. Variability of I content in kelp-based supplements is therefore lower compared to edible seaweed (24). Other algae samples had I content below the limit of detection of XRF method.

### Toxic Elements Content in Algal Dietary Supplements

**Figure 3:**
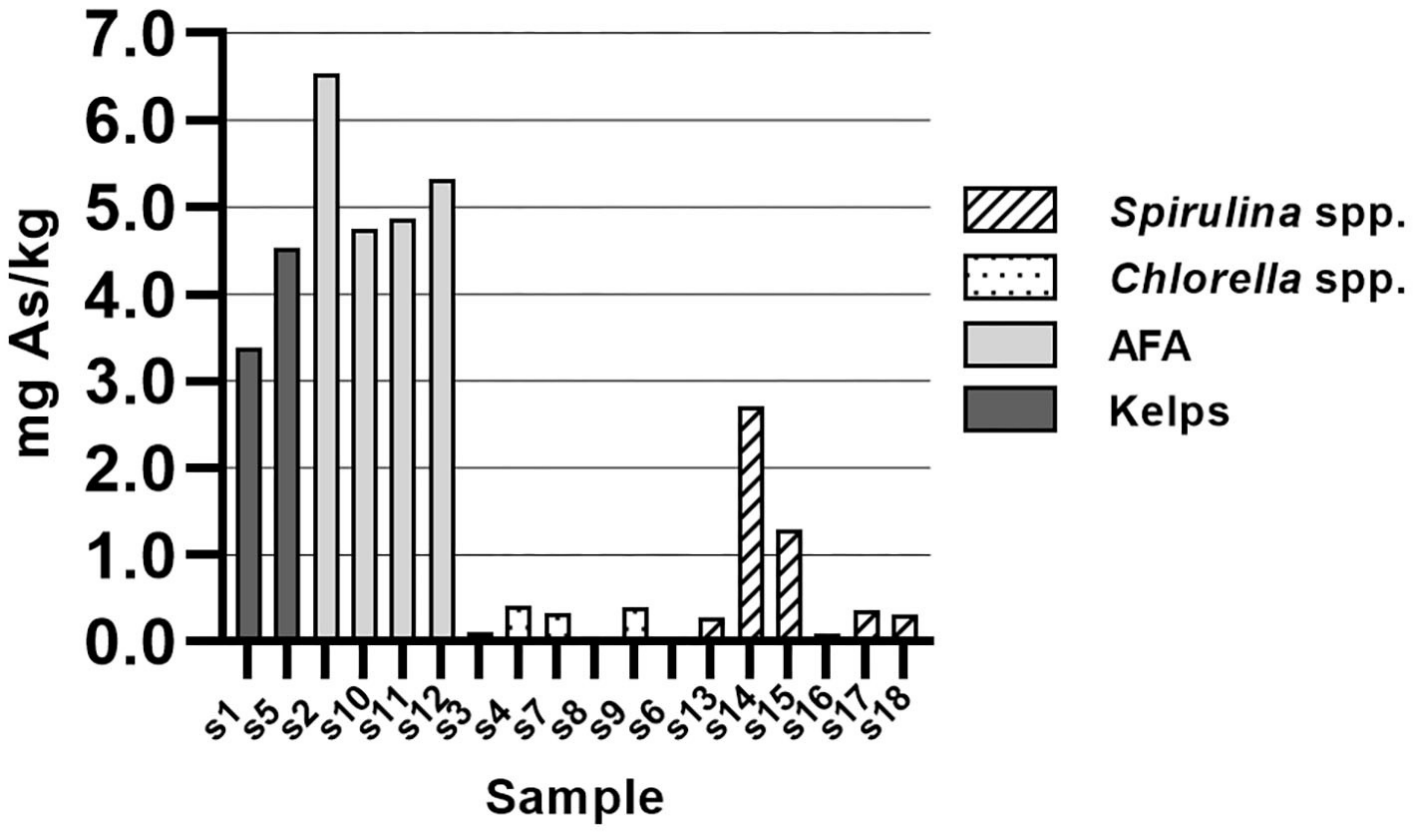
Arsenic content in samples of algal dietary supplements, expressed as mg total arsenic/kg solid sample.

Statistically significant difference in As concentration was found between *Chlorella* spp. and *Spirulina* spp. samples and AFA and kelp-based algae. Average total arsenic (As) content of samples was 0.26 ± 0.17 mg/kg for *Chlorella* spp. and 0.73 ± 0.96 mg/kg for *Spirulina* spp. samples (Figure 3). AFA and kelp-based samples had higher total As content, which was between 3.5 and 6.5 mg/kg solid sample. At the time of writing, European Commission (25) has not set upper tolerable level for As content in dietary supplements. It should be noted that our analysis determined only total As. For health risk assessment, determination of As species is required as the inorganic form of As is more toxic compared to the organic form.

**Figure 4:**
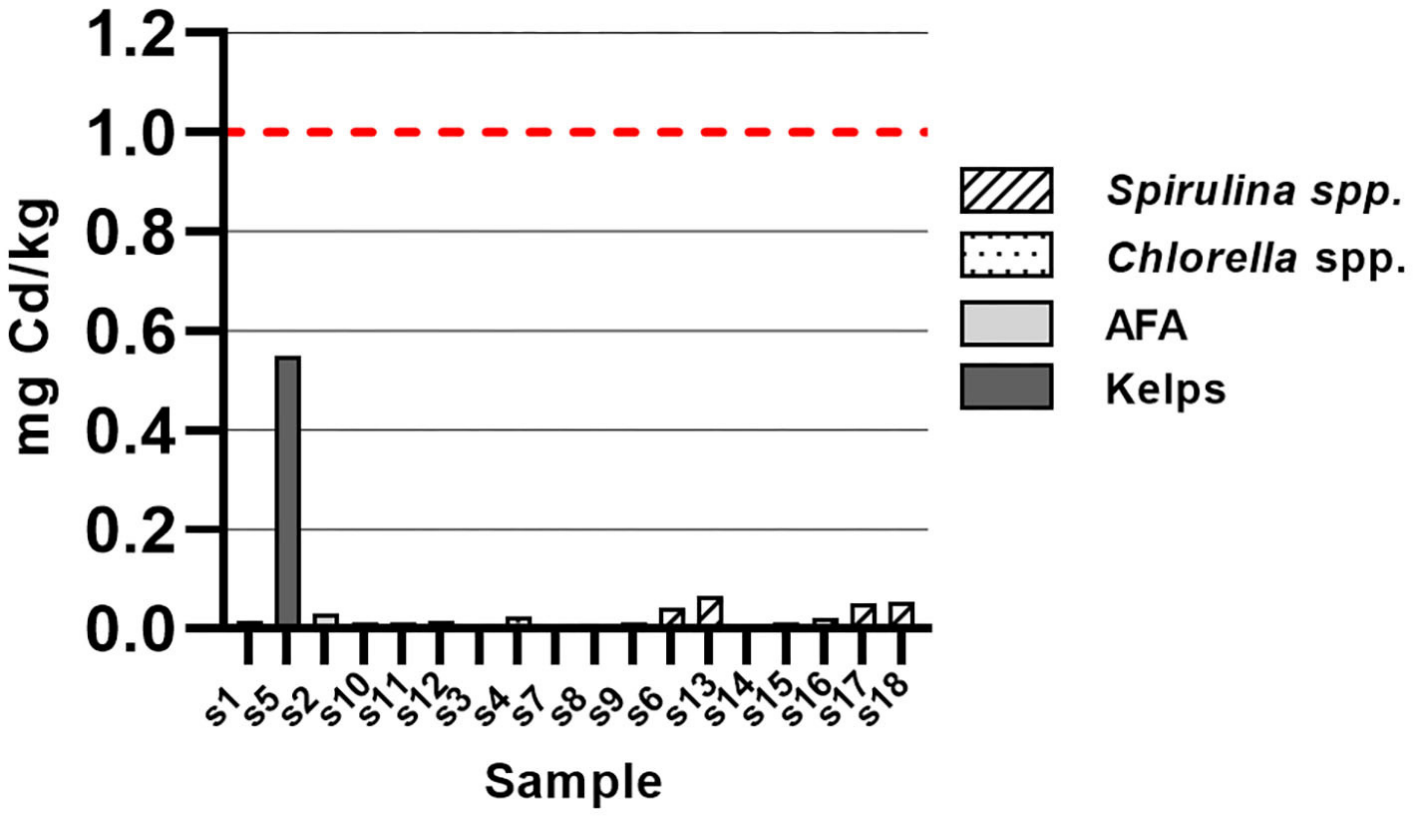
Cadmium content in samples of algal dietary supplements, expressed as mg total cadmium/kg solid sample.

Cd content was below maximum allowed value for dietary supplements of 1.0 mg/kg, set by European Commission (25) (Figure 4). Kelp sample (S5) had notable Cd content of 0.55 mg/kg solid sample and as such differs significantly from other samples. Other samples had Cd content between 0.011 and 0.064 mg/kg. With respect to Cd content, none of the samples pose risk to consumers' health.

**Figure 5:**
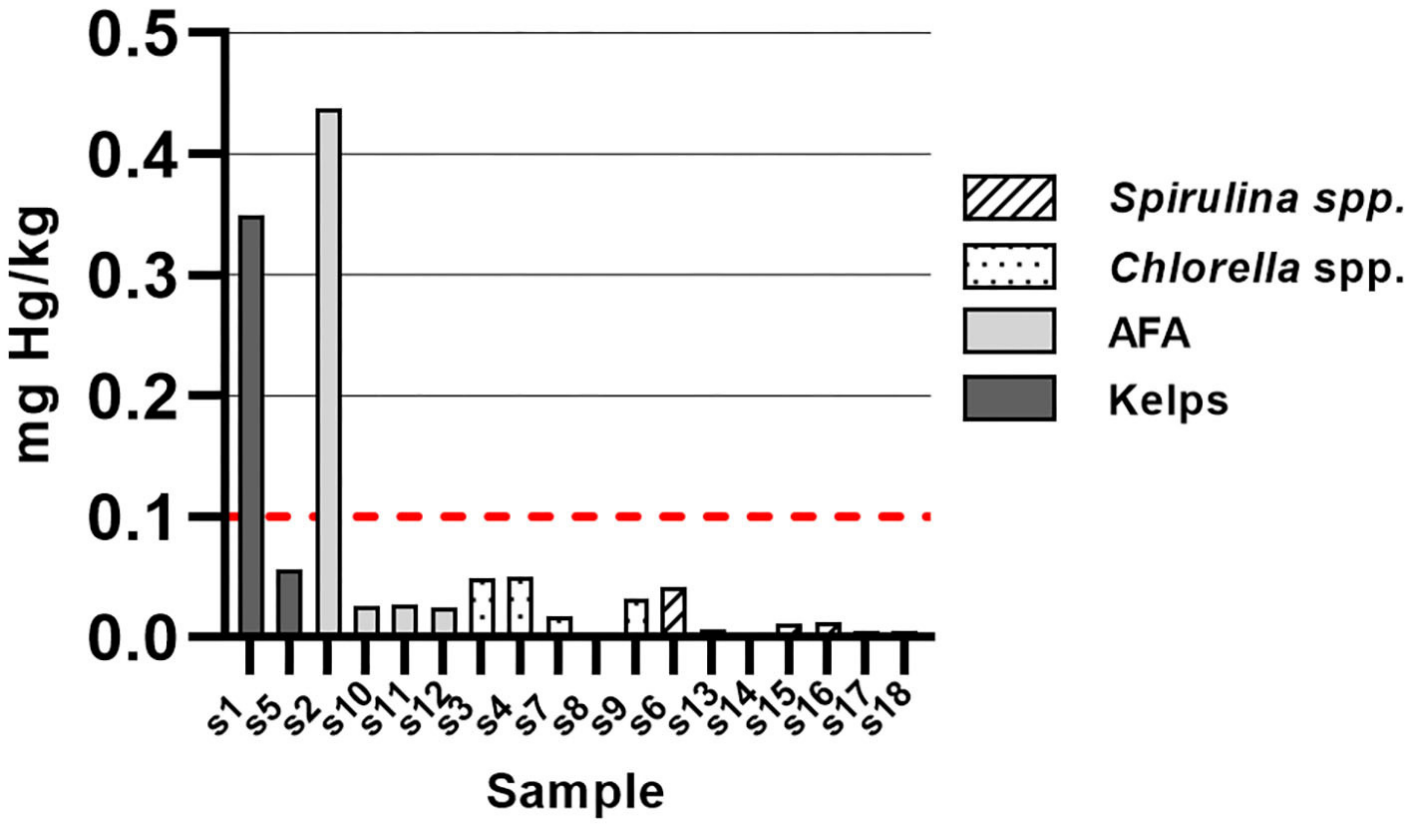
Mercury content in samples of algal dietary supplements, expressed as mg total mercury/kg solid sample.

Samples of kelps (S1) and AFA (S2) exceeded maximum allowed value of Hg in dietary supplements by factor of 3.5 and 4.4, respectively (Figure 5). Maximum allowed value of Hg in dietary supplements is set at 0.1 mg/kg by European Commission (25). Hg content of other samples was below maximum allowed value. One sample of *Chlorella* spp. (S8) had Hg content below LOD. Despite the exceeded values of Hg in two samples, it should be noted that maximum Hg content for fish is set much higher (compared to dietary supplements) at 1.0 mg/kg fish muscle (25). By ingesting manufacturer's recommended daily dose of sample (4.02 g) with highest Hg content, we would ingest 1.76 μg of Hg. In contrast, eating 100 g of fish flesh with Hg content at limit (1.0 mg/kg) would equate to ingesting 0.1 mg Hg, which is 57 times higher than daily dose of sample (S2) with the highest Hg content.

**Figure 6:**
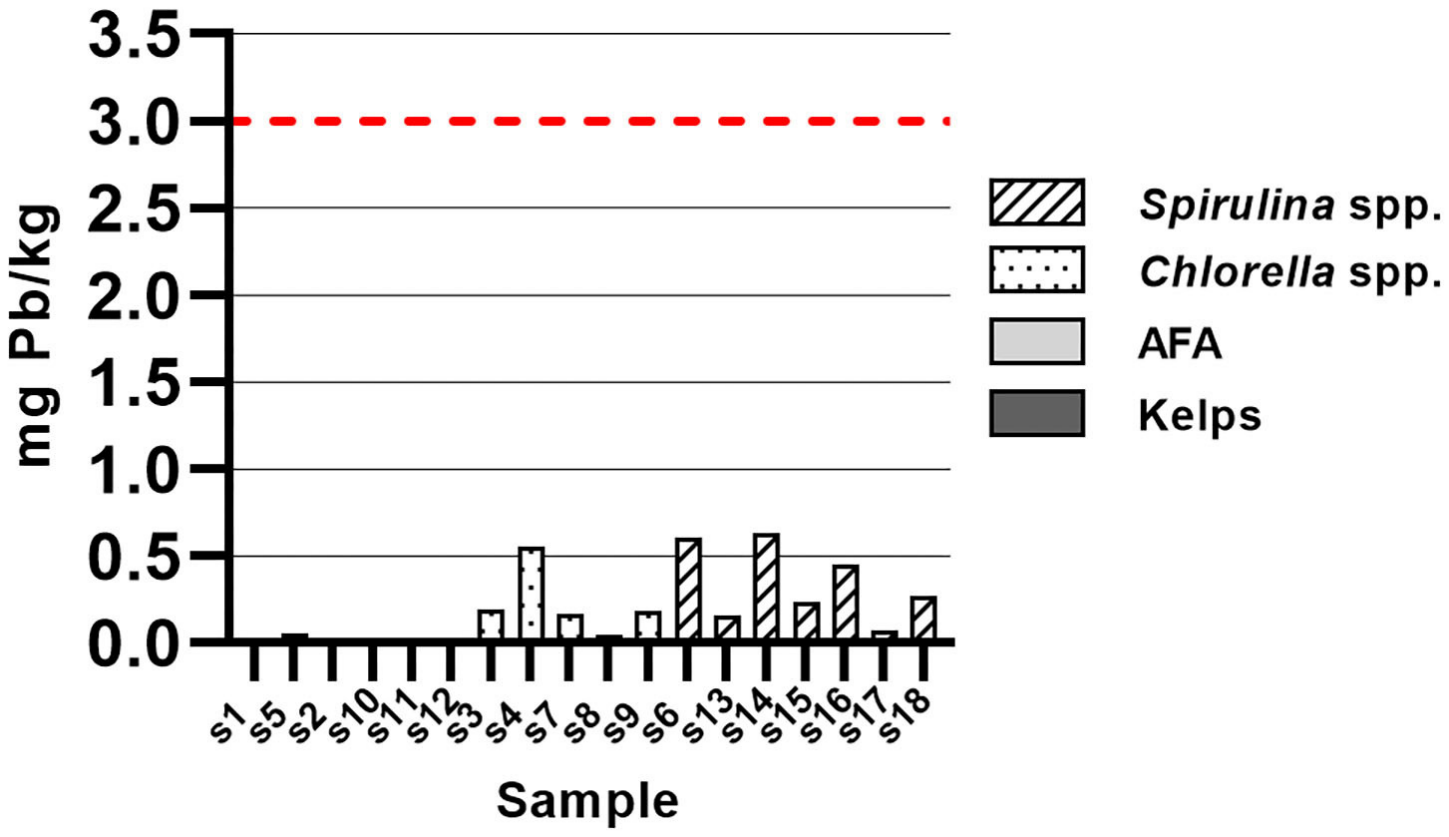
Lead content in samples of algal dietary supplements, expressed as mg total Pb/kg solid sample.

Pb content was below maximum allowed value (3.0 mg/kg) (25) in all samples of algal dietary supplements (Figure 6). Kelps sample (S1) had Pb content below LOD. Pb content of samples was 0.35 ± 0.22, 0.23 ± 0.19, and 0.02 ± 0.00 mg/kg for *Spirulina* spp., *Chlorella* spp., and AFA, respectively, where *Spirulina* spp. differ significantly from AFA samples. In contrast with our results, Rzymski et al. (7) report of average Pb content of 2.6 ± 1.3 mg/kg for *Chlorella* spp. samples and 2.6 ± 1.9 mg/kg for *Spirulina* spp. samples, where 40% of *Chlorella* spp. and 30% of *Spirulina* spp. samples exceeded maximum allowed value.

Toxic elements such as Hg, Cd, Pb, and As were detected only in trace amounts and as such do not pose risk to consumers' health, which is in agreement with other studies (7, 10). It should be noted that algal products also require determination of algal toxins to fully evaluate their safety (6).

### PCA of Microalgae Samples

**Figure 7:**
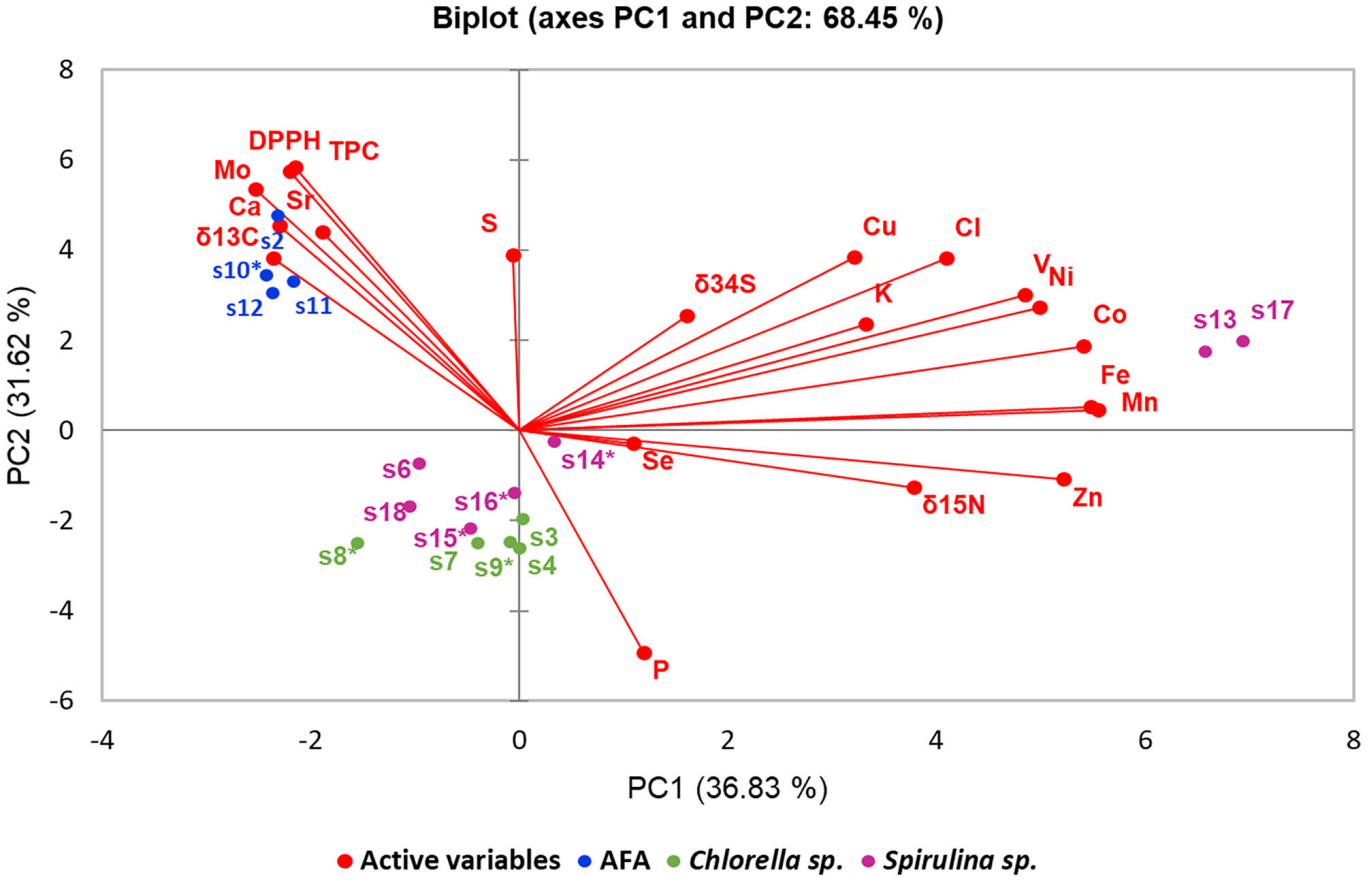
Principal component analysis of algal dietary supplements analysis results (antioxidative potential, elemental, and stable isotope composition data). The biplot shows data of samples as dots (scores) and analysis results (loadings) as vectors. Samples marked with * are declared as organic.

AFA-based products are distinguishable from other samples (Figure 7). They have characteristically high antioxidative potential (TPC and DPPH); high Mo, Ca, and Sr content; low P content; relatively low δ^15^N values; and high δ^13^C values. Sample S2 slightly deviates from AFA group, possibly due to additives (all other AFA samples are declared as pure). Based on our PCA, we can claim that AFA samples originate from the same source, supposedly Klamath Lake, OR. Interestingly, sample S10 is declared as organic, whereas other samples have no such declaration. Such labeling discrepancy is unexpected, considering all our AFA samples are advertised to originate from Klamath Lake.

Samples of *Chlorella* spp. and *Spirulina* spp. cannot be reliably distinguished using PCA (with exception of Hawaiian *S. pacifica*) because of lack of characteristic parameters of respective microalgae (Figure 7). Organically grown *Spirulina* spp. and *Chlorella* spp. also do not exhibit any characteristic parameters, including δ^15^N values, where high values usually indicate assimilation of organic N originating from wastewater. Two *S. pacifica*. samples (S13 and S17), originating from Hawaii, are well separated from other samples based on Fe, Mn, Zn, Co, Ni, V, K, Cl, Cu, and δ^15^N values and δ^34^S values, whereas the sample from Italy (S18) cannot be distinguished from other *Spirulina* spp. samples originating from non-EU countries.

Hawaiian *S. pacifica* samples S13 and S17 (Figure 7) significantly differ from other analyzed samples. That is largely due to significantly higher content of elements such as Co, Mn, Fe, Ni, V, and Zn compared to other samples, which is shown by their respective loadings (Figure 7).

By combining results from stable isotope composition, antioxidative potential, and elemental composition, we can reliably discriminate *S. pacifica* and AFA from our samples. Discrimination between *Chlorella* spp. and *Spirulina* spp. is not possible based on our results because of insufficient number of samples and scarce information provided by the manufacturers.

## Data Availability Statement

The raw data supporting the conclusions of this article will be made available by the authors, without undue reservation.

## Author Contributions

NO, NP, and JK: Conceptualization. JK, NP, NO, and JM: Methodology. MJ, JK, and MN: Validation. JK, MN, and MJ: Formal analysis. JK, MJ, JM, and MN: Investigation. NP, NO, and MN: Resources. JK: Writing—original draft preparation and visualization. JK, MJ, NO, NP, and MN: Writing—review and editing. NP and NO: Supervision, project administration, and funding acquisition. All authors contributed to the article and approved the submitted version.

## Conflict of Interest

The authors declare that the research was conducted in the absence of any commercial or financial relationships that could be construed as a potential conflict of interest.
