# Supplementary material for: Characterization of Algae Dietary Supplements Using Antioxidative Potential, Elemental Composition, and Stable Isotopes Approach
Source: Front Nutr. 2021 Feb 5;7:618503. doi: 10.3389/fnut.2020.618503 (PMC7892597; doi:10.3389/fnut.2020.618503)
Supplement: Supplementary file 1 [file Table_1.DOCX]

Supplementary Material

# Supplementary Data

**Supplementary Table 1.** Mean concentration (Mean) ± standard deviation (SD), minimum (Min) and maximum (Max) of elements in different algae food supplement samples (mg element/kg solid sample) determined by XRF (Br, Ca, Cl, Fe, K, Mn, P, Rb, S, Si, Sr, Ti, Zn) and ICP-MS (As, Cd, Co, Cu, Hg, Mn, Mo, Ni, Pb, Se, Sr, V, Zn) methods.

|  | ***Spirulina* spp. (n = 7)** | | | ***Chlorella* spp. (n = 5)** | | | ***Aphanizomenon* *flos-aquae* (n = 4)** | | | **Kelp (n = 2)** | | |
| --- | --- | --- | --- | --- | --- | --- | --- | --- | --- | --- | --- | --- |
|  | **Mean ± SD** | **Min** | **Max** | **Mean ± SD** | **Min** | **Max** | **Mean ± SD** | **Min** | **Max** | **Mean ± SD** | **Min** | **Max** |
| **As** | 0.73 ± 0.96 | 0.05 | 2.70 | 0.26 ± 0.17 | 0.04 | 0.41 | 5.38 ± 0.81 | 4.76 | 6.54 | 3.96 ± 0.81 | 3.39 | 4.54 |
| **Br** | 5.57 ± 7.80 | 0.50 | 17.40 | 0.93 ± 0.51 | 0.55 | 1.80 | 3.34 ± 1.46 | 1.51 | 4.77 | 81.85 ± 58.19 | 40.70 | 123.00 |
| **Ca** | 2551 ± 3178 | 737 | 9610 | 1970 ± 1396 | 1090 | 4430 | 9048 ± 3805 | 6430 | 14700 | 1178 ± 937 | 515 | 1840 |
| **Cd** | 0.04 ± 0.02 | 0.01 | 0.06 | 0.01 ± 0.01 | 0.00 | 0.02 | 0.02 ± 0.01 | 0.01 | 0.03 | 0.28 ± 0.38 | 0.01 | 0.55 |
| **Cl** | 2367 ± 2613 | 92 | 5770 | 219 ± 236 | 82 | 638 | 2045 ± 574 | 1340 | 2630 | 6420 ± 4950 | 2920 | 9920 |
| **Co** | 3.41 ± 5.36 | 0.16 | 13.14 | 0.63 ± 0.34 | 0.10 | 1.00 | 0.70 ± 0.21 | 0.40 | 0.90 | 0.05 ± 0.01 | 0.05 | 0.06 |
| **Cu** | 3.85 ± 3.52 | 0.83 | 9.97 | 3.28 ± 1.98 | 1.94 | 6.75 | 5.31 ± 0.63 | 4.51 | 5.99 | 0.32 ± 0.16 | 0.21 | 0.44 |
| **Fe** | 1360 ± 1334 | 281 | 3480 | 996 ± 524 | 544 | 1900 | 438 ± 49 | 377 | 491 | 126 ± 6 | 122 | 130 |
| **Hg** | 0.01 ± 0.01 | 0.01 | 0.04 | 0.03 ± 0.02 | * | 0.05 | 0.13 ± 0.21 | 0.03 | 0.44 | 0.20 ± 0.21 | 0.06 | 0.35 |
| **K** | 13904 ± 5003 | 5830 | 20800 | 9528 ± 1310 | 7570 | 11100 | 11800 ± 1538 | 10000 | 13700 | 4465 ± 3500 | 1990 | 6940 |
| **Mn**^1^ | 69.0 ± 63.1 | 19.3 | 185.0 | 43.7 ± 16.1 | 16.7 | 55.8 | 21.5 ± 3.4 | 17.6 | 25.4 | 8.4 ± 0.4 | 8.2 | 8.7 |
| **Mn**^2^ | 65.4 ± 59.2 | 15.4 | 169.0 | 40.6 ± 14.9 | 16.1 | 51.2 | 19.1 ± 0.6 | 18.6 | 19.7 | 2.6 ± 0.3 | 2.4 | 2.8 |
| **Mo** | 0.30 ± 0.24 | 0.12 | 0.77 | 0.24 ± 0.07 | 0.18 | 0.36 | 4.86 ± 0.77 | 4.11 | 5.83 | 0.09 ± 0.04 | 0.07 | 0.12 |
| **Ni** | 1.96 ± 1.93 | 0.22 | 5.10 | 0.40 ± 0.12 | 0.19 | 0.51 | 1.03 ± 0.35 | 0.61 | 1.47 | 0.33 ± 0.04 | 0.30 | 0.35 |
| **P** | 11137 ± 2495 | 6160 | 13600 | 14460 ± 1742 | 12800 | 16700 | 6095 ± 814 | 4940 | 6800 | 538 ± 575 | 131 | 944 |
| **Pb** | 0.35 ± 0.22 | 0.08 | 0.63 | 0.23 ± 0.19 | 0.05 | 0.55 | 0.02 ± 0.00 | 0.02 | 0.03 | 0.03 ± 0.03 | * | 0.06 |
| **Rb** | 4.47 ± 4.59 | 0.55 | 11.90 | 2.38 ± 1.21 | 0.87 | 3.82 | 2.26 ± 0.63 | 1.47 | 2.99 | 3.00 ± 3.11 | 0.80 | 5.20 |
| **S** | 7203 ± 1651 | 3880 | 9040 | 6638 ± 740 | 5870 | 7730 | 8683 ± 1215 | 7470 | 10100 | 980 ± 750 | 449 | 1510 |
| **Se** | 0.55 ± 0.97 | 0.02 | 2.70 | 0.05 ± 0.03 | 0.02 | 0.10 | 0.11 ± 0.02 | 0.08 | 0.12 | 0.01 ± 0.01 | 0.01 | 0.02 |
| **Si** | 10699 ± 6630 | 1340 | 21700 | 8962 ± 6122 | 2840 | 15600 | 3790 ± 3075 | 2120 | 8400 | 1175 ± 7 | 1170 | 1180 |
| **Sr** ^1^ | 21.0 ± 17.0 | 7.4 | 56.2 | 15.8 ± 12.8 | 7.9 | 38.5 | 55.2 ± 29.2 | 36.1 | 98.7 | 79.1 ± 63.6 | 34.1 | 124.0 |
| **Sr**^2^ | 19.1 ± 16.7 | 6.1 | 54.3 | 19.5 ± 12.8 | 7.9 | 37.2 | 52.0 ± 29.6 | 32.2 | 96.0 | 80.2 ± 64.0 | 34.9 | 125.4 |
| **Ti** | 20.0 ± 16.8 | 8.8 | 46.5 | 33.8 ± 45.6 | 8.3 | 115.0 | 6.8 ± 1.3 | 5.5 | 8.5 | 5.7 ± 2.6 | 3.9 | 7.6 |
| **V** | 1.93 ± 2.98 | 0.08 | 7.31 | 0.23 ± 0.16 | 0.11 | 0.50 | 1.19 ± 0.74 | 0.44 | 2.21 | 0.47 ± 0.04 | 0.44 | 0.50 |
| **Zn**^1^ | 22.3 ± 16.1 | 6.0 | 52.7 | 18.5 ± 9.1 | 8.2 | 31.2 | 3.7 ± 1.1 | 2.6 | 5.0 | 1.1 ± 0.3 | 0.9 | 1.3 |
| **Zn**^2^ | 26.0 ± 18.4 | 10.2 | 60.5 | 24.1 ± 13.7 | 10.4 | 41.7 | 3.8 ± 1.1 | 2.8 | 5.3 | 1.4 ± 0.5 | 1.1 | 1.8 |
| ^1^Measured by XRF.  ^2^Measured by ICP-MS. *Below limit of detection of the method. | | | | | | | | | | | | |
